# Household Hints and Recipes

**Published:** 1879-04

**Authors:** 


### Household Hints & Recipes.

—A tablespoonful of ammonia in one gallon of warm water will restore the color of carpets.

A CHEAP AND GOOD PREPAPED GLUE.—Dissolve common glue in cider vinegar as thick as may be wanted ; as it becomes thick from time to time add vinegar.

—IRIDESCENT glasses are now made by burning chloride of tin in the furnace. Fumes are thus produced for which warm glass has great affinity, and which immediately produce an iridescent surface upon it. To heighten the tints a small quantity of the nitrates of baryta and strontia may be used.

TO WHITEN ORGAN OR PIANO KEYS.—Make a thin paste of spirit of ammonia and Spanish whiting, and rub them thoroughly with the mixture. When dry, wipe off the powder with a flannel rag. The ammonia removes the stains, and the whiting gives to the ivory a beautifully white appearance.

TO WHITEN SMOKY CEILINGS.—*J. Platt, Jr.* (*Lexington, Ky.*) recommends to use English prepared whiting mixed with water and a little alum. Lime should not be used, as the smoke will show through it. He says he has used the above for his houses during twenty years, and was always complimented by all for the whiteness of his ceilings.

TO PRESSRVE FLOWERS FRESH.—In the winter season, when flowers are rare and costly, it is desirable to keep them from perishing as long as possible. They may be kept fresh for from fifteen days to a month by inserting their stems in water in which salamoniac has been dissolved in the proportion of twenty-five grains to the the quart.

WHITE WASH.—Whitewash should be applied as often as once a year to cellars, outbuildings, and to rough board fences that can not be painted. Take a lump of lime and slake it with boiling water ; cover it during the process ; strain it, and add a little salt dissolved in warm water, half a pound of Spanish whiting, two ounces of glue. This is good for ceilings, walls, wood, brick, or stone.

STEEL RUST.—According to the *Chemiker Zeitung*, articles of steel which have become rusty may be cleansed by brushing with a paste made up of 30 parts of cyanide of potassium, 30 parts curd soap, 50 parts of precipitated chalk, and a sufficiency of water. Great care is required in preparing and using this poisonous mixture.

FURNITURE POLISH.—Alcohol, 21 ounces ; gum shellac, 2 ounces ; linseed oil, 14 ounces ; gum benzoin, 2 ounces ; oxalic acid, 1 ounce ; white resin, 2 ounces. Dissolve the gums and acid in the alcohol, let it remain 24 hours and then add the oil. This polish has been in use in the writer's family for fully 50 years, in a damp climate, and has been found to keep the furniture in excellent condition.

IRON THAT WILL NOT RUST.—Prof. Barff has discovered that if iron be subjected to the action of steam having a temperature of 1,500° F., it is covered by an incorrodible coating of magnetic oxide, giving the finished article a dull black appearance, susceptible of a slight polish. Salt or fresh water, vegetable acids, and all other ordinary oxidizing agents, have had no effect on the iron prepared by Barff's process. It should be called "Barff's iron," after the inventor.

WRITING PENCILS FOR GLASS.—

Stearin, 20 parts.
Suet, 15 parts.
Beeswax, 10 parts.

Melt and add finely powdered

Red lead, 30 parts.
Dry carbonate of potash, 5 parts.

Let stand in a warm place, stirring occassionally, for one hour, and pour out in glass tubes.

IMPRESSIONS FROM PRINTS.—The print is first soaked in a solution of potash, and then in one of tartaric acid ; this produces a perfect diffusion of crystals of bitartrate of potash through the texture of the unprinted part of the paper. As this salt repels oil, the ink roller may now be passed over the surface without transferring its contents to the paper, except in those parts to which the ink has been originally applied. The ink of the print prevents the saline matter from penetrating wherever it is present, and wherever there is no saline matter present the ink adheres, so that many impressions may be taken, as in lithography.

